# Obesity and Type 2 Diabetes mellitus induce lipopolysaccharide tolerance in rat neutrophils

**DOI:** 10.1038/s41598-018-35809-2

**Published:** 2018-12-03

**Authors:** Wilson Mitsuo Tatagiba Kuwabara, Caroline Naomi Fukusawa Yokota, Rui Curi, Tatiana Carolina Alba-Loureiro

**Affiliations:** 1 Department of Physiology and Biophysics, Institute of Biomedical Sciences, University of São Paulo, São Paulo, Brazil; 2 Interdisciplinar Health Science Post-Graduate Program, Cruzeiro do Sul University, São Paulo, Brazil

## Abstract

Obesity and diabetes implicate in various health complications and increased mortality caused by infection. Innate immune system is broadly affected by these diseases, leading the patients to an immunosuppressive state. A mechanism that leads innate immune cells to a less capacity of killing microorganism is the impaired TLR4 activation. TLR4 recognizes a component of the outer membrane of Gram-negative bacteria, lipopolysaccharide (LPS), and when activated increases the production of inflammatory substances. Neutrophils are components of the innate immune system and are the first responders to an invading agent. The correct activation of TLR4 in these cells is required for the initiation of the inflammatory process and elimination of the microorganisms. The aim of this study was to evaluate the influence of type 2 diabetes and obesity in the TLR4 pathway in rat neutrophils. Two experimental models were used: Goto-Kakizaki rats and high-fat-diet induced obese Wistar rats. To evaluate neutrophil response to LPS, intratracheal LPS instillation was used. Neutrophils from obese and diabetic animals exhibited tolerance to LPS, mainly by the impaired production of cytokines and chemokines and the low content of phospho-NFκB and phospho-IKBα. Neutrophils from both experimental models had increased cell death, impaired *in vivo* migration and myeloperoxidase activity.

## Introduction

Obesity and type 2 diabetes mellitus (T2DM) are associated to worsen outcomes and mortality caused by infection^1–6^. The main cause of this vulnerability is the impaired innate immune functions in these two conditions, such as phagocytosis^7,8^, cytokine and reactive oxygen species (ROS) production^8–10^, bactericidal activity^8,11^ and chemotaxis^8,12^. Innate immune system plays an important role in the initiation of inflammation and also in the activation of the adaptive immunity^13^. An imbalance in the innate immune system does compromise the inflammatory process. Innate immune cells are the first responders to invading microorganisms and are capable to recognize specific molecular patterns, such as pathogen-associated molecular patterns (PAMPs) and danger-associated molecular patterns (DAMPs), activate specific intracellular pathways and trigger the production of cytokines and chemokines that enhance the capacity to fight against the invader^14^.

PAMPs and DAMPs are recognized by pattern recognition receptors (PRRs), which are broadly expressed by the innate immune cells. PRRs are divided into four major groups: Toll-like receptors (TLR), RIG-I-like receptors, NOD-like receptors, and C-type lectin receptors^13^. The TLR family is well characterized by 10 members in humans and 12 in mice^15^. Each of the TLR members recognizes a specific molecular pattern, allowing the immune cells to respond specifically against a wide range of microorganisms, such as bacteria^16^, viruses^17^ and fungi^18^. TLR4, for instance, recognizes lipopolysaccharide (LPS), a component of Gram-negative bacteria, which is known to cause septic shock when its concentration is elevated in the blood^13^. The balance between activation and inhibition of the TLR pathway plays a key role to avoid chronic inflammatory process and correct elimination of the microorganism^19^. However, some diseases may disturb this dynamic balance, letting the individual more susceptible to infections or development of autoimmune diseases^20^.

Diabetes and obesity may affect the TLR4 activation pathway in some innate immune cells, such as monocytes and macrophages^21^. Neutrophil is the most abundant circulating innate immune cell in humans^22^ and are the first cell line that achieves the site of inflammation^22^. Neutrophils, as the other innate immune cells, express most of the TLR members and respond promptly when these receptors are activated^23^. The influence of diabetes (type 1 and 2) and obesity in neutrophil’s function is well-characterized^24–28^. However, how these diseases affect the TLR pathways in neutrophils remains to be elucidated. The aim of this study was then to evaluate the influence of T2DM and obesity in the TLR4 pathway of neutrophils from rats. Herein, two different experimental models were used: the spontaneous type 2 diabetic rats Goto-Kakizaki and high fat diet (HFD) induced obese Wistar rats. To assess neutrophils response to LPS, an *in vivo* intratracheal LPS instillation model was used.

In this study, it was demonstrated that neutrophils from diabetic and obese animals are tolerant to LPS. Neutrophil TLR4 pathway was affected in the two experimental models, resulting in impaired production of cytokines and chemokines, migration and myeloperoxidase (MPO) activity.

## Results

### Characteristics of the experimental models

Metabolic characterization of GK and HFD induced obese rats was previously published in Kuwabara *et al*.^29^. Control and 60% high fat diets were given to the animals for 8 weeks. GK rats are spontaneous type 2 diabetic animals obtained from the Wistar lineage by repetition of selective breeding^30^. After 8 weeks of diet administration, GK rats presented all the classical features of T2DM such as insulin resistance, fasting hyperglycemia, hyperinsulinemia and increased levels of plasma triglycerides and cholesterol^29^. On the other hand, HFD failed to induce T2DM in Wistar rats. However, HFD fed animals presented liver fat accumulation, postprandial glucose intolerance, white adipose tissue (WAT) insulin resistance, and inflammation^29^. Thus, GK and HFD fed rats are two different experimental models with two different metabolic profiles, i. e., GK rats as a non-obese T2DM model and HFD fed animals as obese rats with important metabolic alterations.

### Neutrophil migration, cytokine/chemokine production and MPO activity

**Figure 1:**
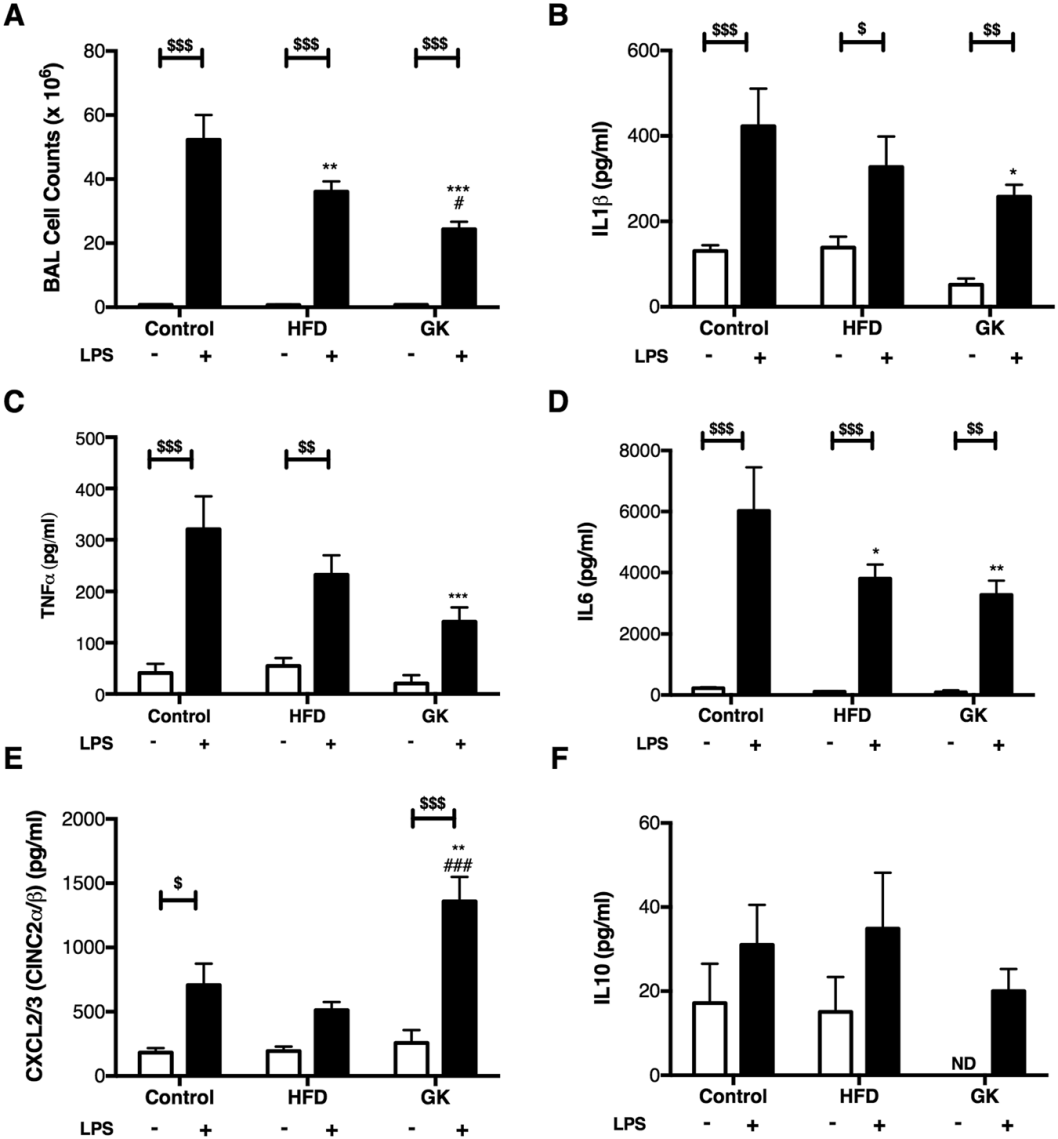
Number of cells (**A**) and cytokine/chemokine (**B–F**) production in the BAL of the different groups. Cells were counted in a Neubauer chamber and cytokines were measured by ELISA. Control (n = 11); HFD (n = 9); GK (n = 10). Results are presented as mean ± S.E.M and n represents the number of animals used in each group. *p < 0.05 *vs* control; **p < 0.01 *vs* control; ***p < 0.001 *vs* control; ^#^p < 0.05 *vs* HFD; ^###^p < 0.001 *vs* HFD; ^$^p < 0.05 *vs* saline instillation of the same studied group; ^$$^p < 0.01 *vs* saline instillation of the same studied group; ^$$$^p < 0.001 *vs* saline instillation of the same studied group. ND (Not detected).

**Figure 2:**
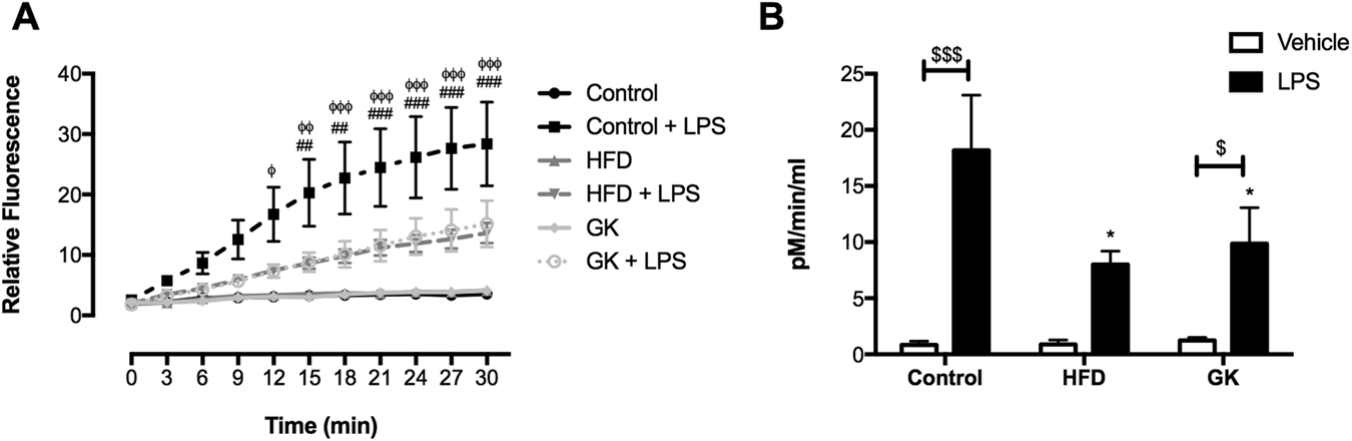
MPO activity of the BAL after vehicle or LPS intratracheal instillation. Control (n = 10), HFD (n = 9) and GK (n = 13). Results are presented as mean ± S.E.M and n represents the number of animals used in each group. *p < 0.05 *vs* control; ^##^p < 0.01 *vs* HFD; ^###^p < 0.001 *vs* HFD; ^ϕ^p < 0.05 *vs* GK; ^ϕϕ^p < 0.01 *vs* GK; ^ϕϕϕ^p < 0.001 *vs* GK; ^$^p < 0.05 vehicle *vs* LPS; ^$$$^p < 0.001 vehicle *vs* LPS.

In order to evaluate neutrophil response to LPS, neutrophil count, cytokines/chemokines measurement, and MPO activity were performed in the BAL of the different groups. LPS intratracheal instillation promoted neutrophil migration to the lungs and same time saline instillation did not stimulate neutrophil influx (Fig. S1). The Number of neutrophils collected from the BAL was lower in GK and HFD fed rats (Fig. 1A). Decreased contents of IL1β, IL6, TNFα and increased of CXCL3 were found in the BAL supernatant of GK rats whereas in the HFD group only a decrease in BAL IL6 content was reported (Fig. 1B–F). MPO activity was also measured in the BAL supernatant and results showed that GK and HFD fed rats had lower MPO activity when compared to the control group (Fig. 2). This data demonstrated that neutrophils from GK and HFD groups had an impaired response to LPS.

### Neutrophil death

**Figure 3:**
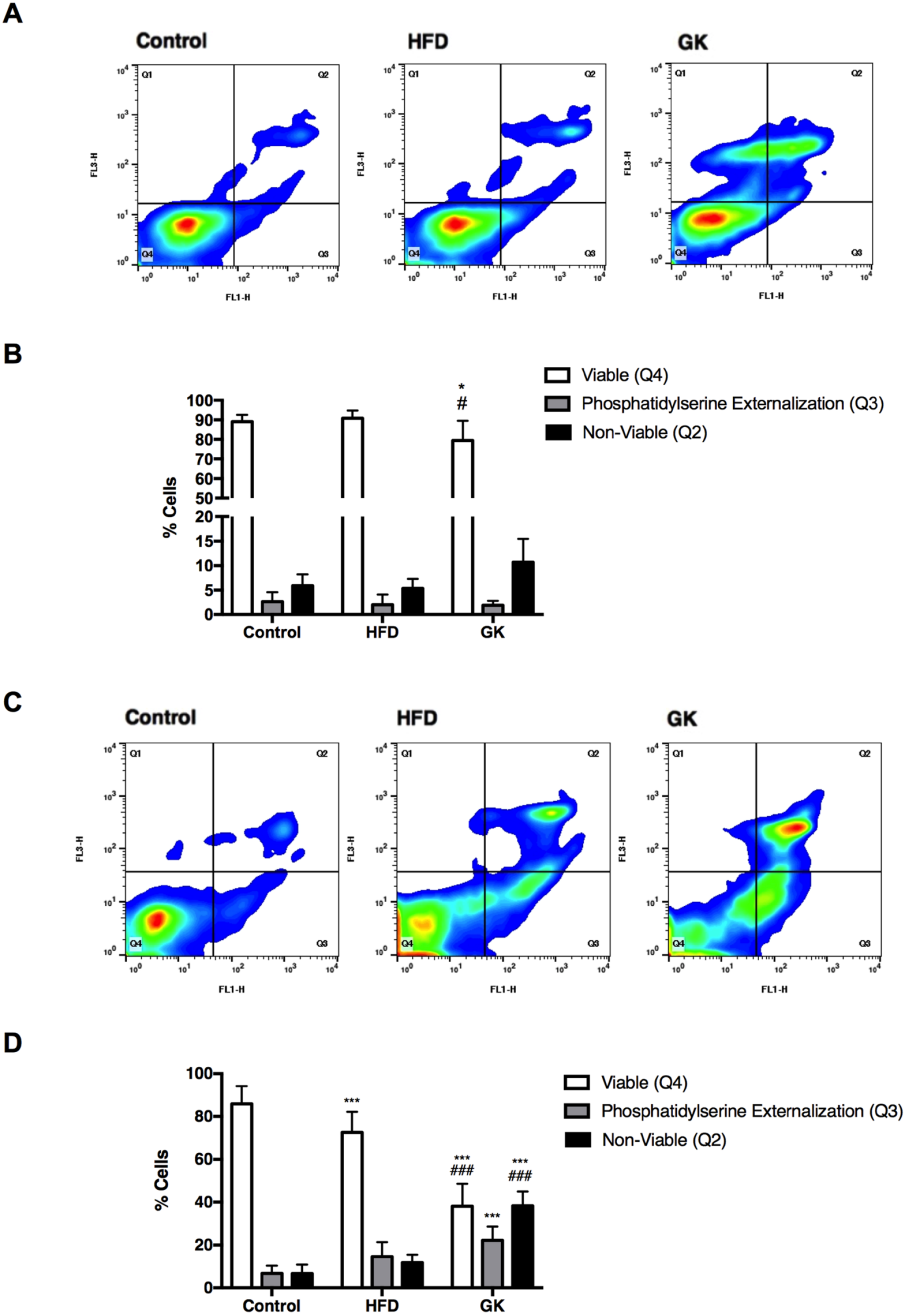
Cell viability and phosphatidylserine externalization in neutrophils collected from the BAL (**A**,**B**) or separated from the blood (**C**,**D**). The dot plot abscissa represents the fluorescence emitted by the annexin V-FITC and the ordinate the fluorescence emitted by the 7-AAD. The Q4 region corresponds to cells with intact plasma membrane; in the Q3 region cells with phosphatidylserine externalized (early apoptotic cells) and in the Q2 region, late apoptotic and necrotic cells. BAL: Control (n = 3), HFD (n = 3) and GK (n = 6); Blood: Control (n = 6), HFD (n = 7) and GK (n = 11). Results are presented as mean ± S.E.M and n represents the number of animals used in each group. *p < 0.05 *vs* control; ***p < 0.001 *vs* control; ^#^p < 0.05 *vs* HFD; ^###^p < 0.001 *vs* HFD.

**Figure 4:**
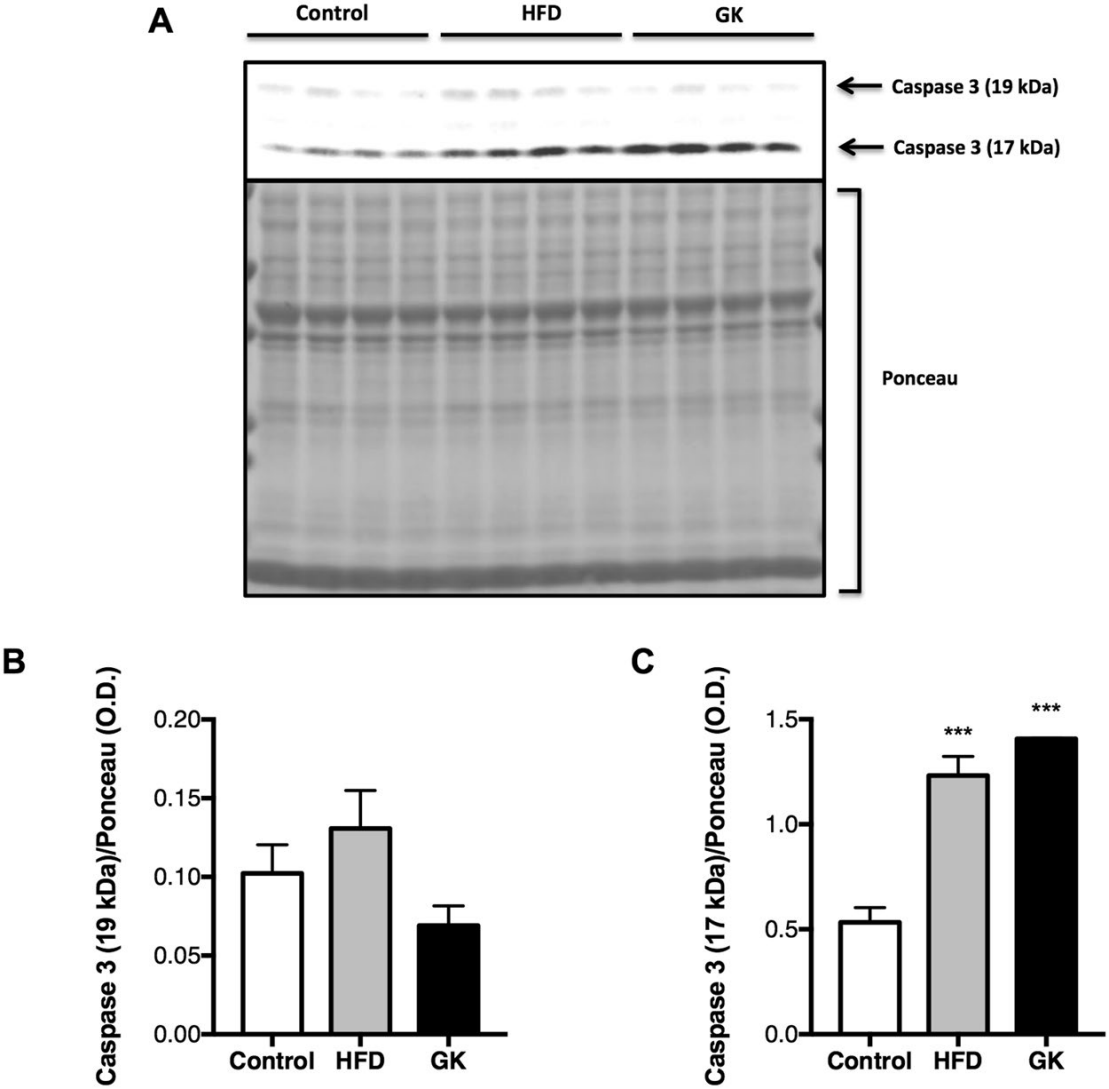
Content of cleaved (17 and 19 kDa) caspase 3 in neutrophils from the blood. Neutrophils were obtained from the blood of the different groups. Graphs represent mean O.D. ± S.E.M of the bands and n represents the number of animals used in each group. Control (n = 4), HFD (n = 4) and GK (n = 4). ***p < 0.001 *vs* control. Ponceau staining of total protein content was used as loading control.

Lower cytokine/chemokine production, MPO activity, and migration could have been caused by early neutrophil death. Thus, neutrophil death was assessed by flow cytometry in neutrophils from the blood (non-stimulated) and from the BAL (stimulated); and by western blotting (cleaved caspase-3 content) in neutrophils from the blood. After LPS stimulus, lower viability was observed in neutrophils collected from the BAL of GK rats (Fig. 3A,B). Neutrophils from the HFD group did not have any difference when compared to the control group (Fig. 3A,B). Analysis of blood neutrophils collected from rats that did not receive any inflammatory stimulus, showed that circulating neutrophils from GK rats had higher incidence of death represented by loss of cell integrity, high externalization of PS and higher content of cleaved caspase-3 (Fig. 4). Neutrophils from the blood of HFD fed rats also demonstrated lower viability (Fig. 3C,D, Fig. 4) and higher content of cleaved caspase-3 (Fig. 4). These results indicate that higher incidence of cell death in circulating neutrophils may be one of the mechanisms that explain the impaired migration and general LPS response in GK and HFD fed rats. Also, the higher rate of death in BAL neutrophils from the GK group may corroborate to the overall impairment cytokine production, which is not observed in the HFD group that only showed decrease in IL6.

### Differential leukocyte count and neutrophil-to-lymphocyte ratio (NLR)

**Figure 5:**
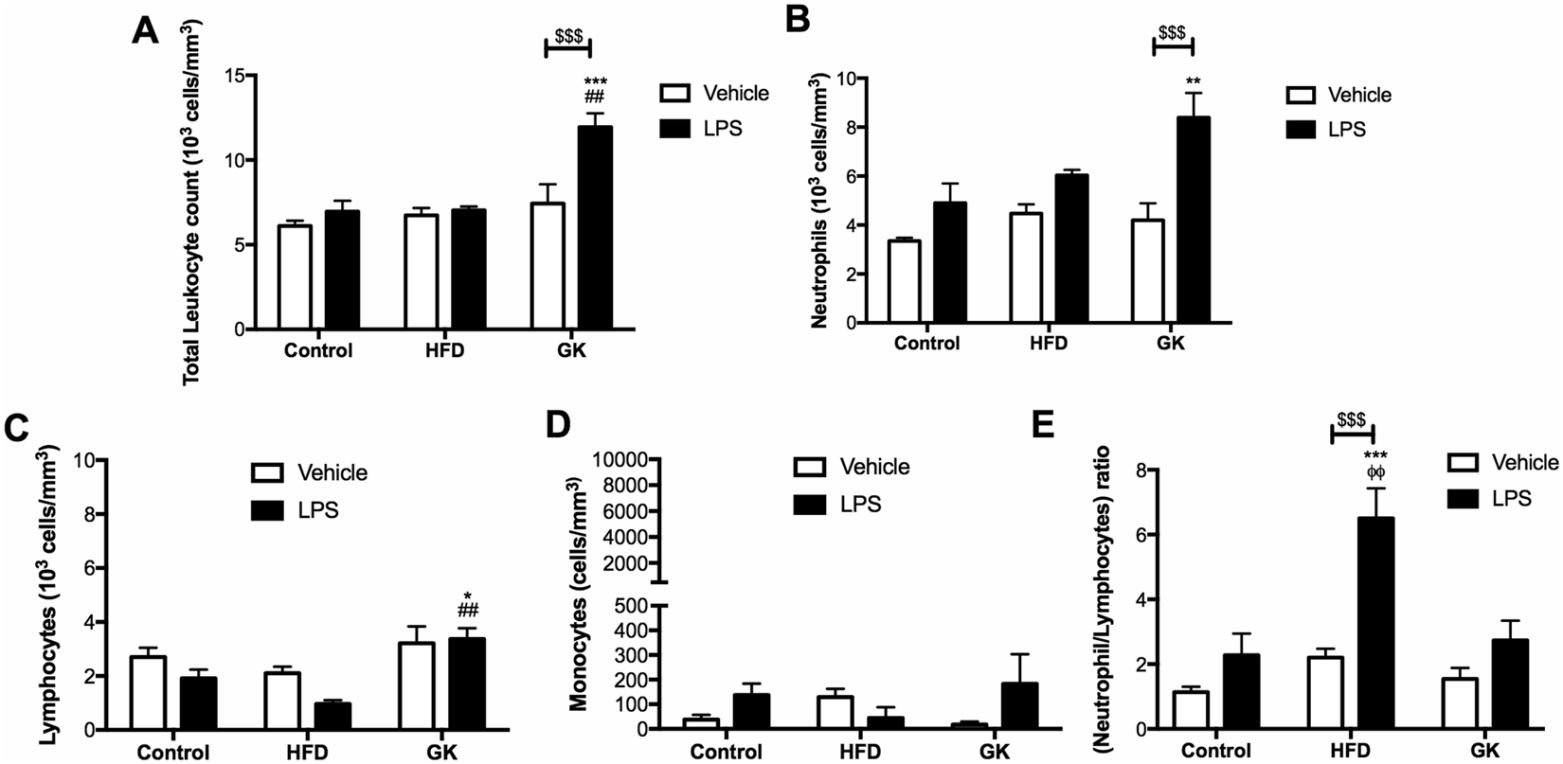
Blood leukocyte count. Blood from the different groups was collected 6 hours after intratracheal vehicle or LPS instillation. Graphs represent Total leukocyte (**A**); Neutrophils (**B**); Lymphocytes (**C**); Monocytes (**D**) and Neutrophil-to-lymphocyte ratio (**E**). Control (n = 7), HFD (n = 5) and GK (n = 7). Results are presented as mean ± S.E.M and n represents the number of animals used in each group. *p < 0.05 *vs* control; **p < 0.01 *vs* control; ***p < 0.001 *vs* control; ^##^p < 0.01 *vs* HFD; ^###^p < 0.001 *vs* HFD; ^$$$^p < 0.001 vehicle *vs* LPS.

Neutrophil death in the circulating blood would possibly change total leukocyte count in GK and HFD fed rats, which would also corroborate to the decreased neutrophil count in the BAL after LPS stimulus. To assess bone marrow capacity to release new neutrophils to the blood stream and verify the leukocyte scenario before and after LPS stimulus, blood leukocyte differential count of the animals was performed. In a non-stimulated state, 10% (6.73 ± 0.45) and 21% (7.42 ± 1.13) augmentation in total leukocyte count was observed in HFD and GK groups, respectively. However, this increase was not statistically different from the control group (6.10 ± 0.32) (Fig. 5A). Also, even though not statistically different, in the basal state, neutrophil count was 25% (4,47 ± 0.37) and 20% (3.68 ± 0.50) higher in the GK and HFD groups respectively, when compared to the control group (3.34 ± 0.13) (Fig. 5B). Increase in total leukocyte and neutrophil counts were observed in the GK group, after LPS stimulus (Fig. 5A,B). Augmented total leukocyte count in this group was mainly due to an increase in circulating neutrophils and lymphocytes (Fig. 5B–D). Increased NLR was higher in the HFD group than in the other groups (Fig. 5E), suggesting that, after LPS stimulus, the myelopoiesis is enhanced over the lymphopoiesis in HFD fed rats. Finally, the results indicate a neutrophil accumulation in the blood after LPS stimulus in the GK group, showing that bone marrow is releasing neutrophils into the bloodstream but these cells are unable to migrate to the site of inflammation.

### Neutrophil gene expression profiles

**Figure 6:**
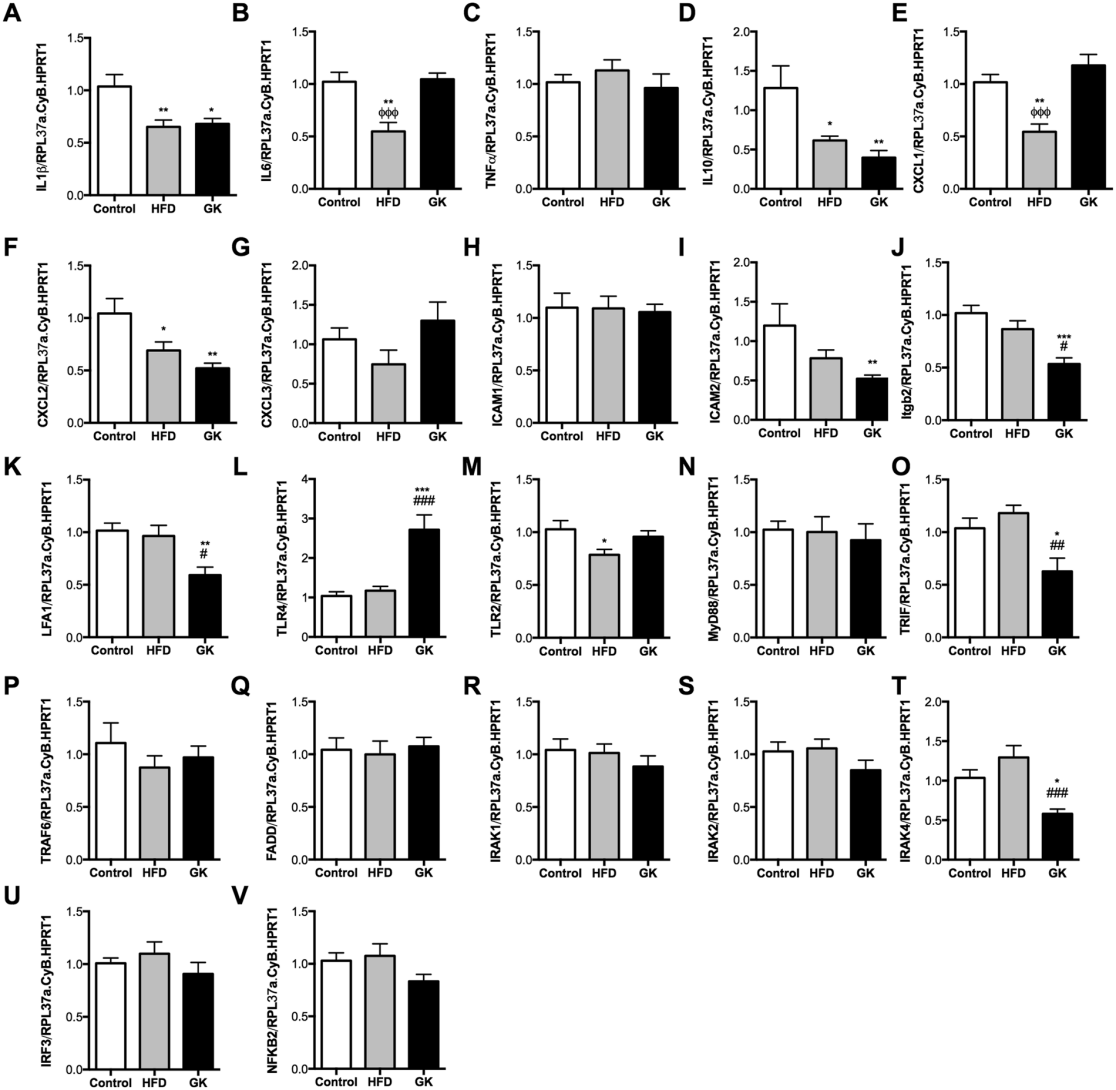
Gene expression profile of cytokines and chemokines (**A**–**G**), adhesion molecules (**H**–**K**) and TLR4 signaling pathway (**L**–**V**). Neutrophils were obtained from BAL 6 h after LPS instillation. Gene expression was evaluated by real-time PCR using SyBR green as a fluorescent probe. Control (n = 7), HFD (n = 8) and GK (n = 7). Results are presented as mean ± S.E.M and n represents the number of animals used in each group. *p < 0.05 *vs* control; **p < 0.01 *vs* control; ***p < 0.001 *vs* control; ^#^p < 0.05 *vs* HFD; ^##^p < 0.01 *vs* HFD; ^###^p < 0.001 *vs* HFD; ^ϕϕϕ^p < 0.001 *vs* GK.

The differences in neutrophil count in the BAL, leukocyte in the blood, neutrophil death, cytokine/chemokine production and MPO activity would indicate the existence of different neutrophil phenotypes in the studied groups. In this context, neutrophil gene expression of cytokines/chemokines, adhesion proteins, and TLR4 pathway proteins was performed. Neutrophils from GK rat BAL, when compared to the control group, had lower expression of IL1β, IL10 and CXCL2 and higher expression of IL6. The expression of adhesion molecules ICAM2, Itgb2 and LFA1 was also lower in the GK group compared to neutrophils from control and HFD fed animals. Neutrophils from diabetic animals had higher expression of TLR4 and lower expression of TRIF and IRAK4 compared to the other groups (Fig. 6A–V). Neutrophils from HFD fed rats had lower expression of IL1β, IL6, IL10, CXCL1, and CXCL2 when compared to healthy animals. No differences in the expression of adhesion molecules and TLR4 pathway intermediates were observed in neutrophils from the HFD group (Fig. 6A–V). These data demonstrated that neutrophils from the GK group were unable to migrate to the site of inflammation due to impaired expression of adhesion proteins after LPS stimulus. Moreover, it was observed that neutrophils from GK and HFD fed rats have different gene expression patterns, which could explain in part some of the differences observed in the inflammatory process.

### TLR4 pathway activation

**Figure 7:**
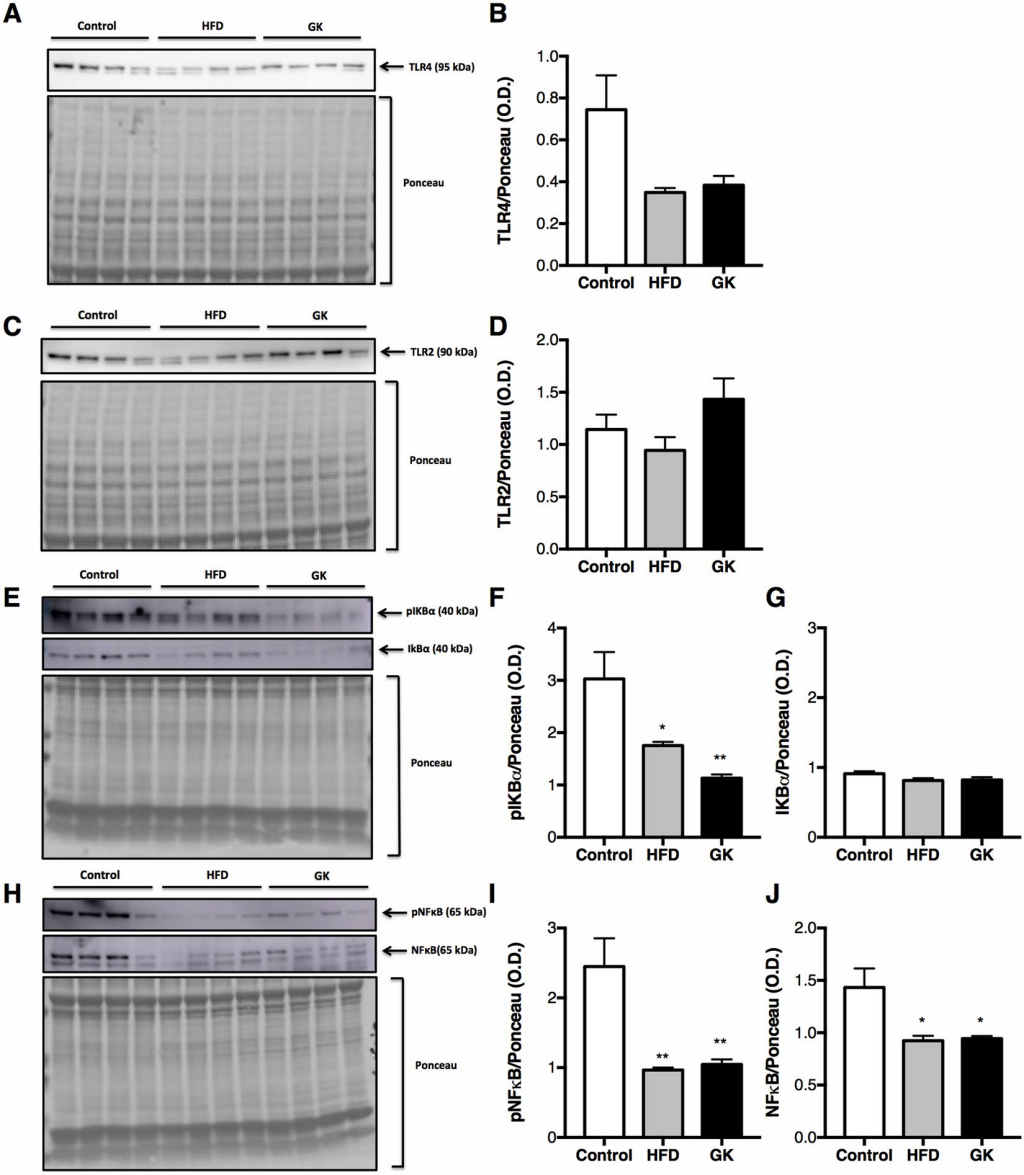
Content of TLR4 (**A**,**B**), TLR2 (C;D;), pIKBα (**E**,**F**), IKBα (**E,G**), pNFκB (**H**,**I**), NFκB (**H**,**J**) in neutrophils from BAL. Neutrophils were obtained from BAL after 6 h LPS instillation. Graphs present mean O.D. ± S.E.M of the bands and n represents the number of animals used in each group. Control (n = 4), HFD (n = 4) and GK (n = 4). *p < 0.05 *vs* control; **p < 0.01 *vs* control. Ponceau staining of total protein content was used as loading control.

To assess neutrophil response to LPS, some key proteins involved in the TLR4 pathway activation was analyzed. Phospho-IKBα, phospho-NFκB and NFκB contents were decreased in neutrophils from the BAL of GK and HFD fed rats (Fig. 7E–J). These results conclude that neutrophils from diabetic and obese rats are LPS tolerant.

### Alveolar macrophages and circulating neutrophil response to LPS (*in vitro*)

**Figure 8:**
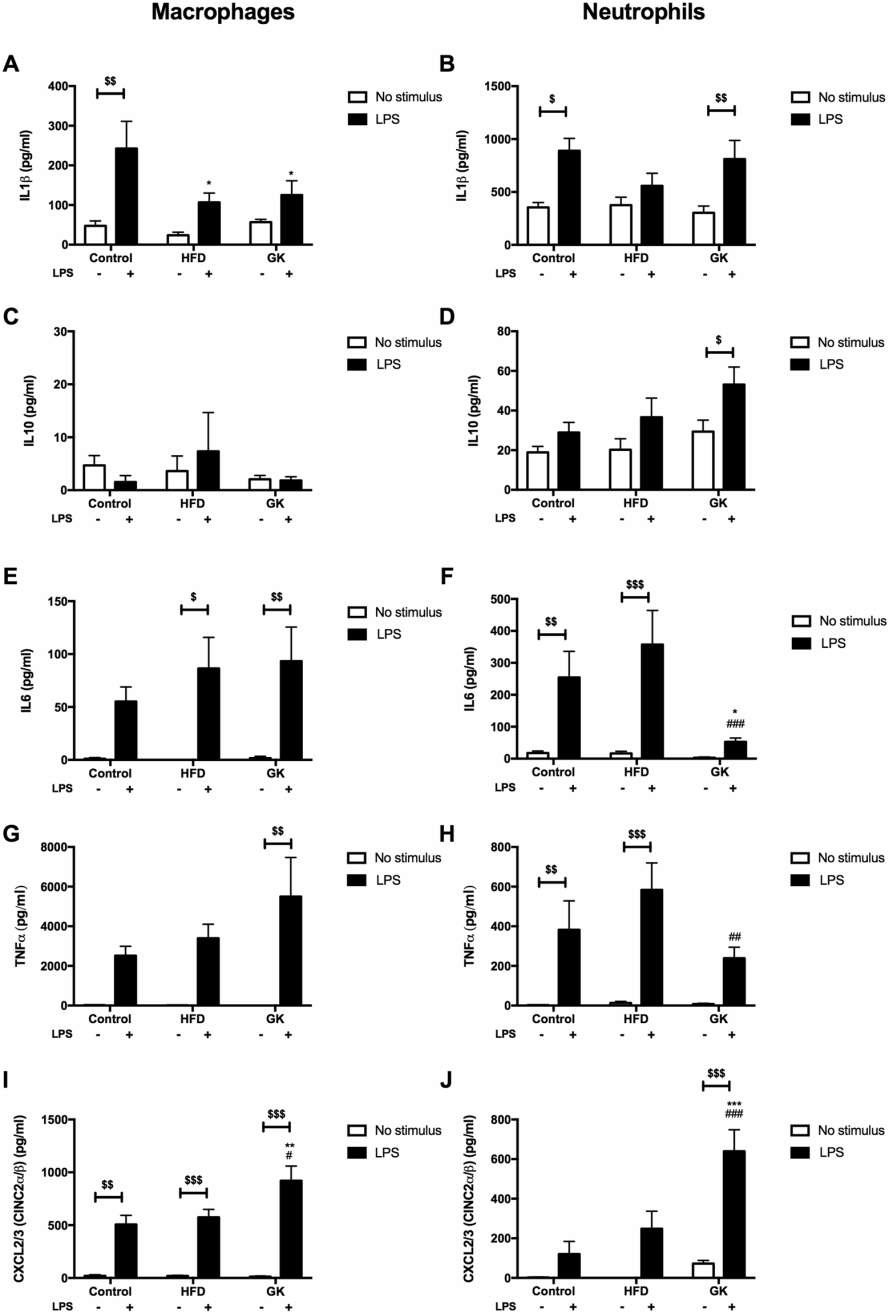
Cytokine production by macrophages (**A,C,E,G,I**) and neutrophils (**B,D,F,H,J**) after *in vitro* 6 h LPS stimulus. Neutrophils were obtained from the blood and macrophages from BAL of the different groups. Macrophages: Control (n = 6), HFD (n = 5) and GK (n = 8); Neutrophils: Control (n = 6), HFD (n = 6) and GK (n = 8) Results are presented as mean ± S.E.M and n represents the number of animals used in each group. *p < 0.05 *vs* control; **p < 0.01 *vs* control; ***p < 0.001 *vs* control; ^#^p < 0.05 *vs* HFD; ^##^p < 0.01 *vs* HFD; ^###^p < 0.001 *vs* HFD; ^$^p < 0.05 vehicle *vs* LPS; ^$$^p < 0.01 vehicle *vs* LPS; ^$$$^p < 0.001 vehicle *vs* LPS.

An *in vitro* LPS stimulation was performed in alveolar macrophages and blood neutrophils to measure their capacity for cytokine/chemokine production independently. Resident population (macrophages) initiates the inflammatory process by attracting neutrophils to the lung after LPS stimulus. To verify the LPS response integrity of this population in the different groups, macrophages were cultured separately for 6 h with or without LPS. Alveolar macrophages from GK rats produced less IL1β and more CXCL3 than macrophages from the control group (Fig. 8A,I). Defective production of IL1β was also observed in macrophages from HFD fed animals (Fig. 8A). Since there was a difference in neutrophil count in the BAL among the groups and this difference affect the final content of cytokines in the BAL supernatant, same number of blood neutrophils from the different groups were cultured and stimulated with LPS to verify their capability of secreting cytokines/chemokines. Thus, this *in vitro* experiment was done in the attempt to eliminate the bias of the different number of neutrophils and different cytokine/chemokine production observed in the BAL between the groups. Neutrophils from GK rats produced less IL6 and TNFα and more CXCL3 than cells from control rats (Fig. 8F–J). Neutrophils from the HFD group did not have any difference when compared to the control (Fig. 8B–J). These results suggest that obesity and T2DM also affect the resident population response to LPS, specifically in the IL1β production. Moreover, in the GK group, the lower cytokine content in the BAL after 6 h LPS stimulus is not determined only by the decreased number of neutrophils, but also by the impaired neutrophil cytokine production. On the other hand, lower content of cytokine in the BAL of obese animals seems to be directly depended on the number of neutrophils that migrated to the lungs.

## Discussion

The immune suppressive state in diabetic and obese patients has been widely described. In ob/ob and diet induced obesity (DIO) experimental models, mice had increased respiratory infections by influenza^31,32^ and bacterial pneumonias^33,34^. Obese mice also had impaired neutrophil chemotactic activity, LPS induced cytokine production and colony-stimulating-factor-mediated survival^35^. In obese humans, neutrophils exhibited elevated ROS production in the basal state and also after stimulus with formyl-methionyl-leucyl-phenylalanine (fMLP) and opsonized zymosan (OZ). Phagocytosis, Cd11b expression, and adherence of neutrophils were not different between obese and lean subjects^24^. In diabetic individuals, neutrophils had spontaneous activation by producing increased levels of ROS in a basal state. However, after stimulus with OZ and phorbol myristate acetate (PMA), neutrophils had impaired production of ROS. Neutrophils also exhibited impaired chemotaxis and cytokine production in diabetic patients^36,37^.

The importance of neutrophils in the inflammation and clearance of the invading microorganism is well documented. However, the mechanisms by which T2DM and obesity impair neutrophil function are still not clear. Neutrophils from spontaneous type 2 diabetic GK and HFD induced obese rats did not respond to LPS as effective as the control animals^29^. We reported that neutrophil TLR4 pathway in GK and HFD fed rats is defective and that this impairment leads to particular alteration in neutrophils from the two different groups. These two experimental models have been characterized in our previous work^29^.

Neutrophils from the HFD group exhibited marked immune dysfunctions. After intratracheal LPS instillation, the number of neutrophils that migrated to the lungs of the HFD fed animals was lower than in the control group, however, no change in neutrophil count in the blood was observed. The difference in neutrophil count between the HFD and control groups may be due to a leak of neutrophils to other inflamed tissues, such as WAT. In fact, an increase in the number of CD11b positive cells in the WAT of HFD fed animals was found^29^. We reported defective production of IL6, decreased expression of IL1β, IL6, CXCL1, CXCL2 and impaired MPO activity in the HFD group. These cytokines and MPO are essential for neutrophil functions and chemotaxis^38^. Since there was no difference in the expression of adhesion proteins, neutrophils from HFD fed animals are likely to have impaired chemotaxis due to a lower expression and content of chemoattractant substances. Indeed, contents of activated intermediates of the TLR4 pathway are lower in the HFD group when compared to control rats.

Obesity paradox is a known medical concept that overweight people are less vulnerable to death in some diseases, such as myocardial infarction^39^, heart failure^40^, renal disease^41^, stroke^42^, and also bacterial and post-surgical infection^43^. This phenomenon is also observed in sepsis^44^, which is an overwhelming immune reaction to infection. Obesity paradox is a really controversial subject in the literature and some recent studies demonstrated that obesity is not associated with a better prognosis in cases of infection and cardiovascular diseases^45,46^. In this context, our results corroborate somehow to this paradox context. Increasing levels of inflammatory cytokines, such as IL1β^47,48^, TNFα^47,48^ and IL6^47–50^ in patients with sepsis are related to worse prognosis and higher rates of death^47–50^. Our data show that obese rats respond less to LPS stimulus, indicating impairment in the TLR4 pathway of these animals’ immune system, which results in less inflammatory cytokines production. One may claim that lower inflammatory cytokines content is related to impaired bacterial clearance, which is true when the inflammatory process is under control; however, in sepsis, there is an overproduction of cytokines that is associated to overall organ dysfunction and consequent death^51^. In this scenario, deficient TLR4 activation in the obese individuals may help to contain the overstimulation and the “cytokine storm” caused by sepsis, improving prognosis and lifespan of obese patients. This same obesity attenuating effect in LPS-induced lung injury was also described in obese mice^52^.

Neutrophils from GK rats had increased rates of cell death when in the blood and also after LPS induced migration to the lungs. Neutrophils death can be triggered by the increased fat and glucose in these animals’ blood and once triggered neutrophils become dysfunctional, unable to migrate and produce cytokines and chemokines^53,54^. In fact, neutrophil count in the BAL of GK rats was lower than in the control group. Concomitantly, an increase in circulating neutrophils after LPS stimulus was found. So, neutrophils are unable to migrate to the lungs, accumulating in the blood possibly because of an impaired response to chemotactic stimulus. GK animals produced less IL1β, IL6, TNFα and CXCL2/3 after LPS stimulus. Neutrophils also had decreased expression of IL1β, IL10, CXCL1, and CXCL2 when compared to the control group. Adhesion proteins, such as LFA1, Itgb2, and ICAM2, were less expressed in neutrophils from GK animals. The main cause of these immune function alterations occurred due to the impaired neutrophil TLR4 activation. Similarly to the HFD fed animals, neutrophils from GK rats had impaired TLR4 pathway activation due to the lower contents of phospho-NFκB and phospho-IKBα. Even though the final inflammatory result after a 6 h LPS stimulus is similar between GK and HFD groups, including less neutrophil migration, low cytokine production and lower content of phospho-NFκB, neutrophils from both groups do not exhibit the same gene expression profile. It is known that metabolic state of the individual may modulate the immune system and the marked metabolic differences between the HFD and GK groups may play an important role on these different neutrophils’ phenotypes.

Diet induced obesity is associated with increased myelopoiesis and leukocytosis in rodent models and humans^55–57^. In fact, a higher NLR was found in HFD fed rats after LPS stimulus. NLR has been used as a marker of bad prognosis in some incident diseases, such as cancer^58^, cardiovascular events^59^, infectious pathologies^60^, obesity^61^ and diabetes^61^. Increased NLR in our HFD model suggests that even though rats did not develop T2DM within 8 weeks of diet administration, the extension of the diet for longer periods would have triggered the syndrome. GK rats showed a great increase in neutrophil and lymphocyte count, which brought NLR values closer to the control group.

Alveolar macrophages and neutrophils from the blood were collected to test *in vitro* LPS activation in these cells separately, unlinking the *in vivo* influence of the pulmonary resident population on the other that migrate due to the LPS stimulus. After LPS stimulus, macrophages from GK and HFD fed rats produced less IL1β than the controls and macrophages from GK rats produced more CXCL3 than cells from the other groups. IL1β induces neutrophil migration by up regulating the expression of adhesion molecules and chemokine production in the local endothelium^62^. Impaired macrophages IL1β production in GK and obese animals contributed to the decreased number of migrating neutrophils in the lungs after LPS stimulus in both groups. CXCL3 is a powerful neutrophil chemoattractant^63^ and both alveolar macrophages and neutrophils from GK rats, when stimulated *in vitro* with LPS, produce more CXCL3 than the other groups. This may indicate again that even though there is a strong signaling for neutrophil migration after LPS instillation, these cells are unable to migrate due to the impaired expression of adhesion molecules on their surface. In summary, the lower content of cytokines and chemokines in the BAL of GK and HFD fed animals after LPS stimulus is due to the lower number of neutrophils that migrated to the lung and the lower expression and production of these substances by neutrophils. Even though *in vitro* blood neutrophils from the HFD group did not show impaired cytokine production; after *in vivo* LPS stimulus, neutrophils that migrated to the lung presented lower content of proteins involved in the TLR4 pathway. This data seems contradictory. However, after an inflammatory stimulus, neutrophils are widely produced in the bone marrow in order to fight the invading microorganisms. Dependently on the organism or metabolic state of the individual, immature or phenotypically different neutrophils are released from the bone marrow to the blood^64–66^. Moreover, it is believed that circulating neutrophils can change their phenotype according to the inflammatory stimulus and metabolic status of the animal^67^. Therefore, we may speculate that after a strong inflammatory stimulus, HFD fed animals failed to produce mature capable neutrophils, releasing immature or phenotypically different neutrophils into the blood. This may explain the difference in the cytokine production, i. e. TLR4-NFκB pathway activation, when blood and BAL neutrophils from the HFD group are compared.

Diabetes and obesity, due to hyperglycemia and high concentrations of plasma free fatty acids, are known to pre-activate leukocytes and lead them to a pro-inflammatory phenotype^24,68–70^. This chronic stimulus by the altered metabolic state in these diseases may lead to the appearance of tolerant or “burned-out” leukocytes that are not able to fully respond to an infectious stimulus, allowing the microorganism to disseminate^71^. We reported for the first time that neutrophils from obese and type 2 diabetic rats are tolerant to LPS. This tolerance may be the explanation for the higher rates of death in obese and diabetic individuals as a consequence of bactericidal infections^33,34^. TLR4 is an essential receptor for the innate immune response and the loss of its pathway activation capacity compromise the inflammatory process. More studies in this area should be performed to elucidate why TLR4 is inactivated in these cells. However, we reckon that elevated plasma levels of insulin of these animals play an important role in suppressing the TLR4 response. Insulin inhibits leukocyte TLR4 signaling in a dose- and time-dependent manner^72^. Since GK and HFD fed rats had increased plasma levels of insulin^29^, this hormone may play a key role for the immunosuppressive state^73^. Endotoxemia is also another possible explanation for this LPS tolerance. High fat diet induces the release of LPS from the gut, even after only a single meal, and diabetic individuals have higher plasma concentrations of LPS^74,75^. So, neutrophils from GK and HFD fed animals are probably regularly exposed to high levels of LPS and insulin. Chronic exposure to LPS also leads to neutrophil LPS tolerance by inducing loss of TLR4 surface expression and consequently defective activation^76^.

## Methods

### Animals

GK and Wistar rats were obtained from Charles River Laboratories International, Inc. (Wilmington, MA, EUA) and maintained in the Institute of Biomedical Sciences of the University of Sao Paulo animal facility. The characterization of these animals was previously published^29^. The Animal Ethical Committee of the Institute of Biomedical Sciences of the University of Sao Paulo (number 109/2013) approved all experimental procedures of this study.

### LPS instillation

Under anesthesia, trachea of the rats was assessed with a catheter and a saline (0.2 mL) containing 750 μg of *E. coli* lipopolysaccharide (Sigma Chemical Co., St. Louis, MO, USA) was instilled into the airways. Control animals received saline only.

### Bronchoalveolar lavage (BAL)

BAL was performed 6 h after the intratracheal LPS or saline instillation. Lungs of the rats were washed with 25 mL phosphate-buffered saline (PBS) at room temperature using a polyethylene tube (1 mm in diameter) inserted into the trachea. Total cell count was determined using a Neubauer chamber and 95% of neutrophil purity was obtained^77^.

### Cell staining

Cells collected from the BAL were adhered onto glass slides by a 5 min, 700 rpm centrifugation using a cytocentrifuge (Excelsa Flex - FANEM). Cells were stained with hematology staining (Instant-Prov kit, Newprov, Paraná, Brazil). Images were taken in a Nikon Eclipse E1000 camera coupled to a light microscopy at 40,000X magnification.

### Blood leucocyte count

Differential count of leucocytes was performed using the equipment ABX Pentra DX 120 (Horiba Medical, Montpellier, France). Blood was collected from rats after 6 h instillation of vehicle or LPS. This equipment uses a Double Hydrodynamic Sequential System technology (Patent of Horiba ABX), which is a combination of Chlorazol black E cytochemistry, focused flow impedance, and light absorbance measurement for blood cell count and 5-part white blood cell differentials^78^.

### Neutrophil separation

Neutrophils were obtained by Ficoll-dextran sedimentation^79^. Briefly, blood samples diluted (1:1) in sterile PBS were layered on an equal volume of Ficoll-Hystopaque (density 1.077 g/mL). After centrifugation (400 × *g*, 30 min, room temperature), the superior mononuclear rich layer was discarded and red blood cells were separated from the neutrophil rich pellet by the addition of 2 mL dextran (6%) for 1 h at 37 **°**C. Neutrophils were identified in a FACScalibur flow cytometer (Becton Dickinson, San Juan, CA, USA) by using the Cell Quest software (Becton Dickinson) and 95% of neutrophil purity was obtained.

### Phosphatidylserine (PS) externalization

PS externalization was analyzed by flow cytometry after PS staining with annexin V according to the method described by Vermes *et al*.^80^.

### *In vitro* LPS stimulus

Neutrophils (1 × 10^6^ cells/mL) obtained from blood and macrophages (2 × 10^5^ cells/mL) obtained from BAL, after saline instillation, were incubated in endotoxin-free RPMI 1640 medium containing 5.5 mM glucose, 10% heat-inactivated FBS at 37°C in a 5% CO_2_ atmosphere for 6 h with or without LPS (2 μg/mL). Supernatant was collected for cytokine and chemokine analysis.

### Determination of cytokine concentrations and MPO activity

The measurements of IL1β, IL6, IL10, CXCL2/3 (CINC-2 α/ β) and TNFα were performed in the BAL fluid and culture supernatant by ELISA using Duo-set available kits (R & D Systems Inc., Minneapolis, MN, USA), following the manufacturer’s instructions. MPO activity was measured in the BAL supernatant using Myeloperoxidase Activity Assay Kit (Fluorometric) (Abcam, Cambridge, UK; ab111749), according to the manufacturer’s instructions.

### Real time polymerase chain reaction (RT-PCR)

Total RNA was obtained from 5 × 10^6^ neutrophils by the guanidine isothiocyanate extraction method using TRIzol® Reagent^81^ (Invitrogen, Carlsbad, CA, USA) followed by isolation using RNeasy mini kit (Qiagen). cDNA was synthesized from total RNA (0.5 µg) using High Capacity cDNA reverse transcription kit (Thermo Scientific), according to the manufacturer’s instructions. Real-time PCR analysis was performed using the SyBR Green JumpStart kit (Sigma Aldrich) in Rotor Gene Q equipment (Corbett Research, Mortlake, Australia)^82^. Gene expression was analyzed by 2^−∆∆CT^ using RPL37a, cyclophilin B (CyB) and HPRT1 as inner controls^83^. Primers of the analyzed genes are exposed in Table S1.

### Western blot analysis

Protein from neutrophils (5 × 10^6^) was extracted in 60 µL Triton X100 lysis buffer containing protease inhibitor cocktail (cOmplete™, Mini, EDTA-free - Roche diagnostics) and PMSF (1 mM). Western blotting was performed according to the protocol used in previous work^40^. Immunoblots were scanned and quantified using ImageJ® software and Ponceau staining was used as an inner control^84,85^. TLR4 and TLR2 polyclonal antibodies were purchased from Santa Cruz Biotechnologies (Dallas, TX, USA). Cleaved caspase-3, pIKBα, IKBα, pNFκB, and NFκB polyclonal antibodies were purchased from Cell Signaling (Danvers, MA, USA).

### Data analysis

Results are presented as mean ± S.E.M. Statistical significance was assessed by two-way ANOVA followed by the Bonferroni post-test. p ≤ 0.05 was considered statistically significant.

### Study approval

Animal studies were approved by the Animal Ethical Committee of the Institute of Biomedical Sciences of the University of São Paulo (number 109/2013). All studies were conducted in accordance with institutional guidelines and regulations at the University of São Paulo.

## Electronic supplementary material

Dataset 1
