# Supplementary material for: Obesity and Type 2 Diabetes mellitus induce lipopolysaccharide tolerance in rat neutrophils
Source: Sci Rep. 2018 Dec 3;8:17534. doi: 10.1038/s41598-018-35809-2 (PMC6277411; doi:10.1038/s41598-018-35809-2)

Obesity and Type 2 Diabetes mellitus induce lipopolysaccharide tolerance in rat neutrophils.

Wilson Mitsuo Tatagiba Kuwabara[*1]; Caroline Naomi Fukusawa Yokota[1]; Rui Curi[1,2]; Tatiana Carolina Alba Loureiro[1]

[1]Department of Physiology and Biophysics

Institute of Biomedical Sciences

University of São Paulo, São Paulo, Brazil.

[2]Cruzeiro do Sul University, São Paulo.

Interdisciplinar Health Science Post-Graduate Program

* Corresponding author:

Wilson Mitsuo Tatagiba Kuwabara

Department of Physiology and Biophysics

1524 Professor Lineu Prestes Avenue, ICB1, 115

University of São Paulo

São Paulo-SP, Brazil

E-mail: wilsonk@icb.usp.br

Phone: +55 11 941422841

+55 11 30917739

Figure S1

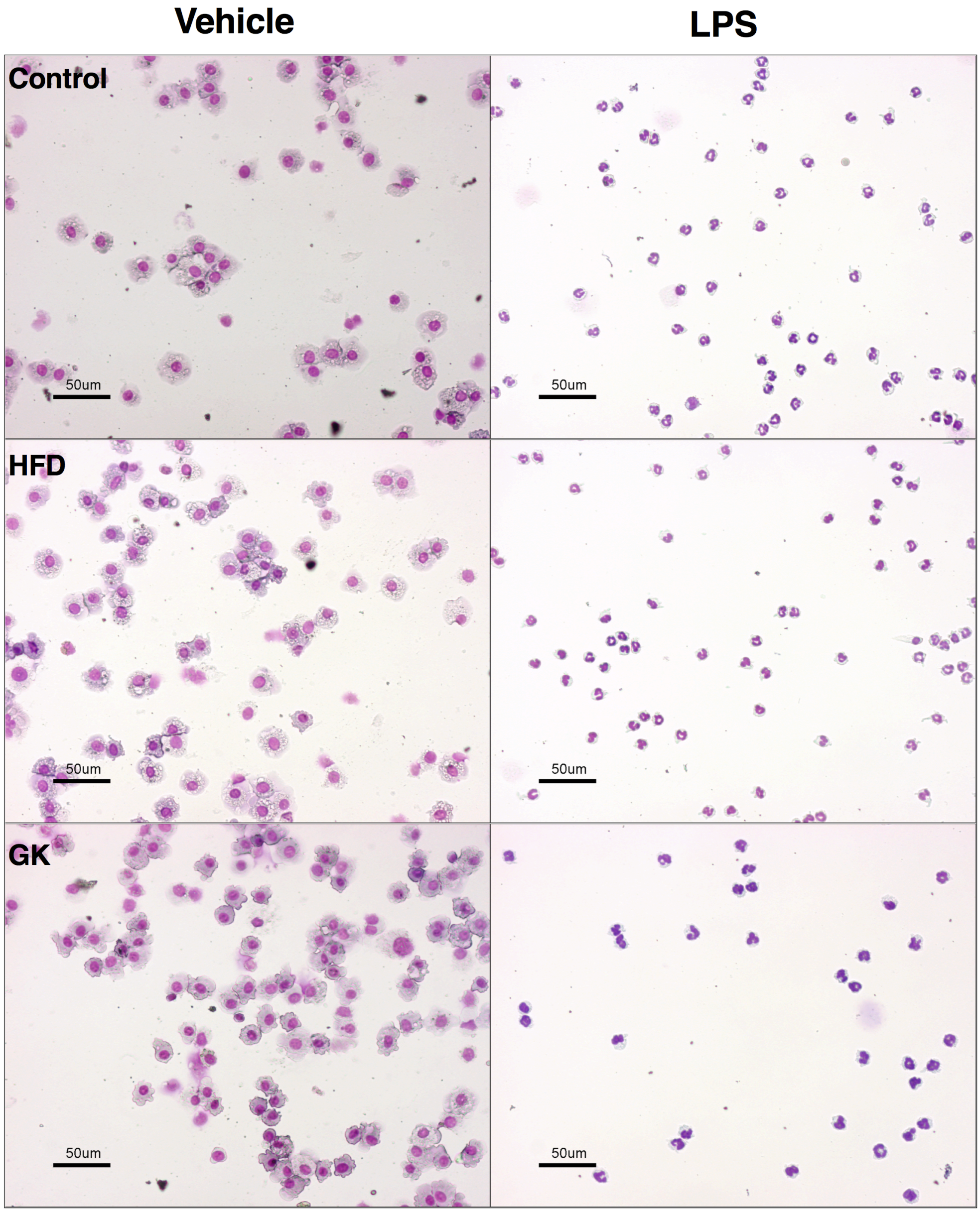

Figure S1. Cells collected from bronchoalveolar lavage (BAL) after vehicle or LPS intratracheal instillation. After 6 hours of vehicle instillation only macrophages were collected from the BAL of all studied groups. LPS stimulus for 6h promoted neutrophil infiltration. Cells were stained with a hematology staining kit. Control (n=6); HFD (n=6) and GK (n=6).

Table S1. Primers sequences, Nm and product length of the genes analyzed.

Table S1

Gene	Primers	Nm	Product lenght
**IL-1β**	S: AAATGCCTCGTGCTGTCTGA<br>AS: AGGCCACAGGGATTTTGTCG	NM_031512.2	133 bp
**IL-6**	S: AGAGACTTCCAGCCAGTTGC<br>AS: AGTCTCCTCTCCGGACTTGT	NM_012589.2	85 bp
**IL-18**	S: AACCGCAGTAATACGGAGCAT<br>AS: CGTTGGCTGTTCGGTCGATA	NM_019165.1	101 bp
**IL-10**	S: CCTCTGGATACAGCTGCGAC<br>AS: ATGGCCTTGTAGACACCTTTGT	NM_012854.2	120 bp
**TNF-α**	S: CTGTGCCTCAGCCTCTTCTC<br>AS: ACTGATGAGAGGGAGCCCAT	NM_012675.3	126 bp
**CXCL-1**	S: CCACACTCAAGAATGGTCGC<br>AS: ACTTGGGGACACCCTTTAGC	NM_030845.1	93 bp
**CXCL-2**	S: CACCAACCATCAGGGTACAGG<br>AS: TTGGTAGGGTCGTCAGGCA	NM_053647.1	71 bp
**CXCL-3**	S:AGCCACTCTCAAGGATGGTCAA<br>AS: ACAGGGAGGGGCTCTTCAGTA	NM_138522.1	95 bp
**LFA-1**	S: ATCGCACACACATGGACTGT<br>AS: GCTTCCCTGGTTGGCTTCTT	NM_01033998	80 bp
**ICAM-1**	S: GCCTGGGGTTGGAGACTAAC<br>AS: CTGTCTTCCCCAATGTCGCT	NM_012967.1	91 bp
**ICAM-2**	S: ATGAGCCTCCAGCTCAGGTA<br>AS: AGAGCAGAGAGAGGGTGAGG	NM_10077251	126 bp
**Itgb2**	S: GTTTCAGACAGAGGTCGGCA<br>AS: AATTTCCTCCGGACAGGCAG	NM_10377802	103 bp
**TLR4**	S: TCCACAAGAGCCGGAAAGTT<br>AS: TGAAGATGATGCCAGAGCGG	NM_019178.1	126 bp
**TLR2**	S: ATCACTGCACCCTCAATGGG<br>AS: TGTGCAGGCTCCGTATTGTT	NM_198769.2	102 bp
**MyD88**	S: GAGAGCAGTGTCCCACAGAC<br>AS: AGCAGATGAAGGCGTCGAAA	NM_198130.1	97 bp
**TRIF**	S: GACTAGAAGTCAGCCTCGCC<br>AS: GCCGGTGGAGTGTAACGTAT	XM_08757917	70 bp
**TRAF6**	S: CGCCAAAATGGAAACGCAGA<br>AS: TGCTTCCATCTCGGCAACTT	NM_01107754	88 bp
**FADD**	S: TGAGGCCAAGATTGATGGCA<br>AS: GAGGCGTTCTCCTTCTCGAC	NM_152937.2	105 bp
**IRAK1**	S: CTCTCCCCAGCTTTTCCAGG<br>AS: GAGGAGGGGGCTGAAGATTG	NM_01127555	107 bp
**IRAK2**	S: ACATCTACCAGCTGCCGTCC<br>AS: TCCCACTCACTGAGCGTGTC	NM_01025422	70 bp
**IRAK4**	S: CCAGTCCAGGCCATCAAAGT<br>AS: CCGATAGACACAGGTCGCTC	NM_01106791	122 bp

**IRF3**	S: GATGAGGGGTCCTCAGACCT<br>AS: TGCTGGGTTTGGGAAGTTGT	NM_01006969	87 bp
**NFKB2**	S: ATGCCGGAGAATGAAGAGCC<br>AS: AGCGTTCAACAGCAGGGTC	NM_00100834	162 bp
**RPL37a**	S:CGCTAAGTACACTTGCTCCTTCTG<br>AS: GCCACTGTTTTCATGCAGGAAC	NM_00120531	91 bp
**CyB**	S: CTCCGTGGCCAACGATAAGA<br>AS: AGGTCACTCGTCCTACAGGT	NM_022536.2	95 bp
**HPRT1**	S: GCGAAAGTGGAAAAGCCAAGT<br>AS:GCCACATCAACAGGACTCTTTAG	NM_012583.2	98 bp

Full length Blots (Western Blot Figure 8)

Ponceau Staining

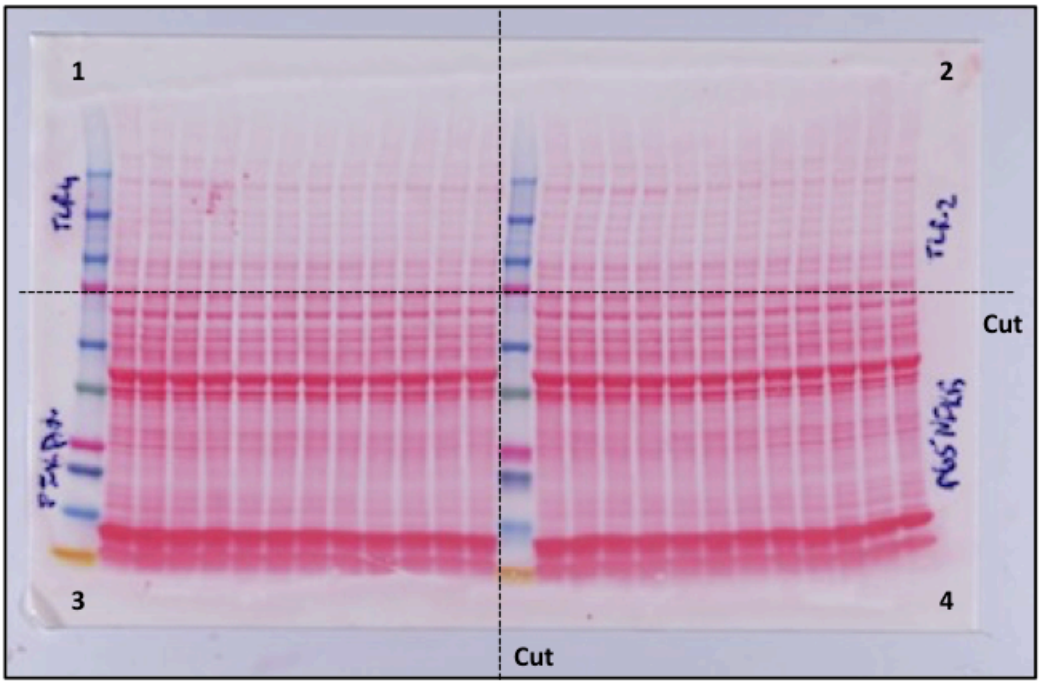

Membrane was cropped in four membranes in order to incubate with the specific antibodies. For NFKB and IKBa, membranes 3 and 4 were stripped and incubated with the specific antibodies.

TLR4

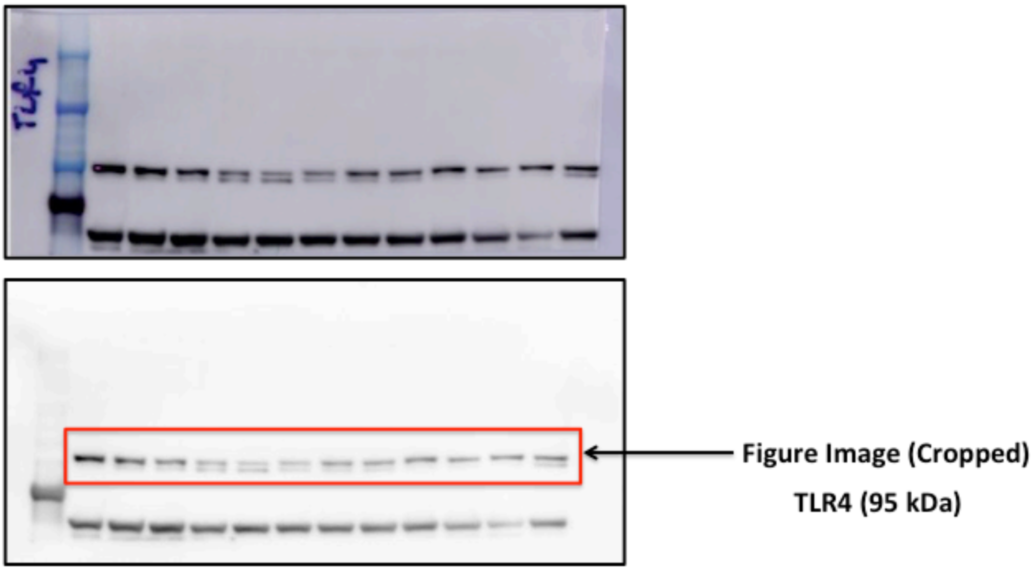

Figure Image (Cropped)
TLR4 (95 kDa)

TLR2

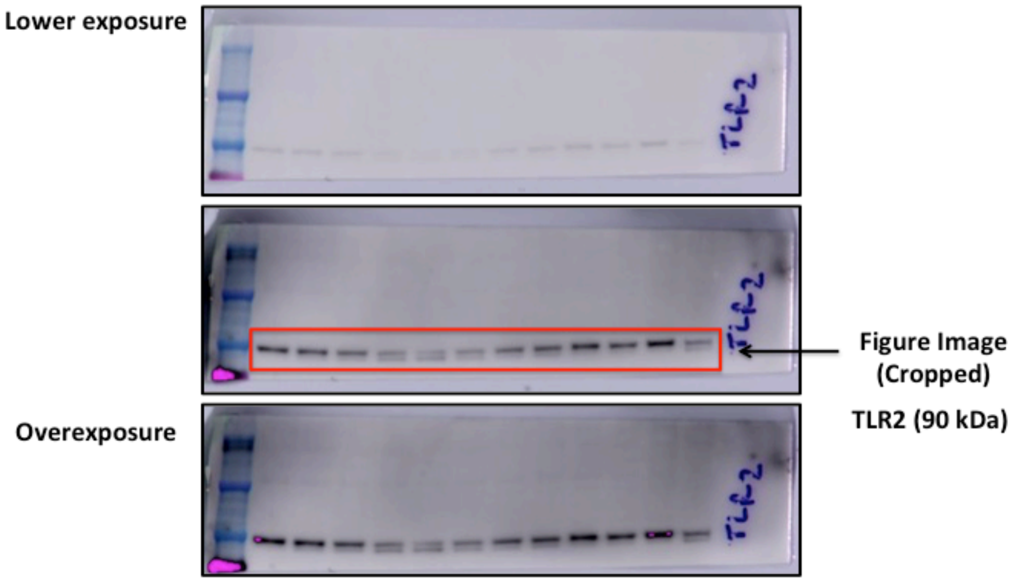

Lower exposure
Figure Image
(Cropped)
TLR2 (90 kDa)
Overexposure

pIKBα/IKBα

Lower exposure

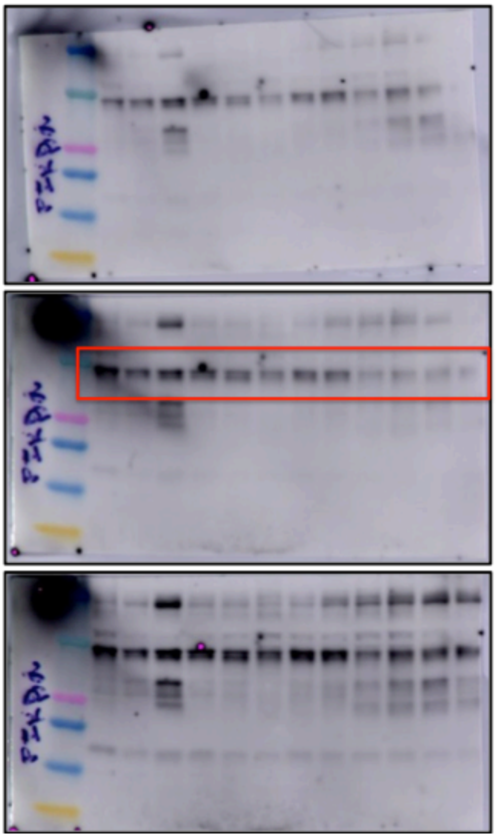

Figure Image (Cropped)

phospho-IKBα (40 kDa)

Overexposure

Lower exposure

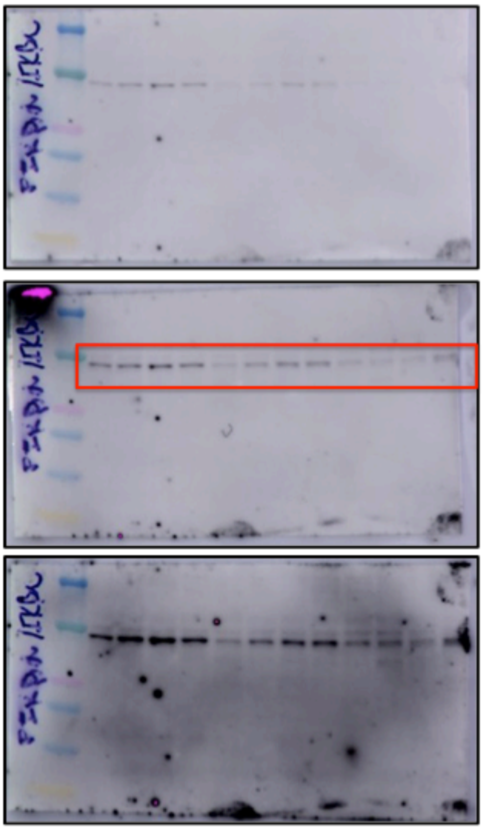

Figure Image (Cropped)

IKBα (40 kDa)

Overexposure

pNFκB/NFκB

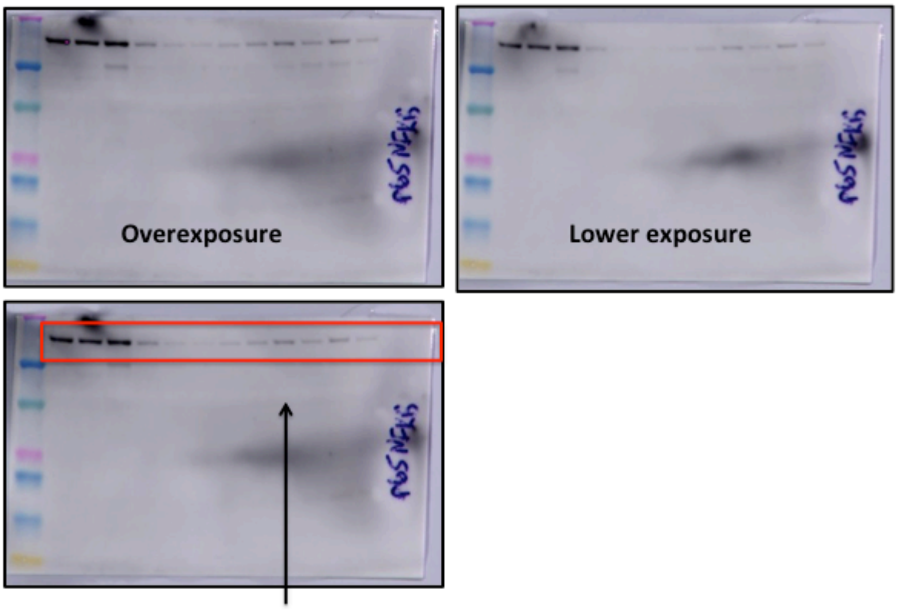

Figure Image (Cropped)
Phospho-NFκB (65 kDa)

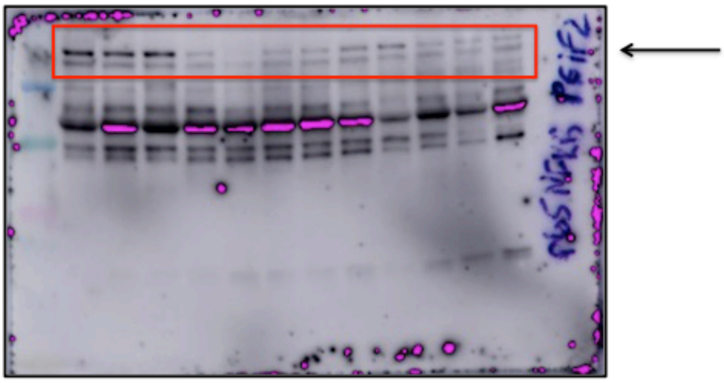

Figure Image
(Cropped)
NFκB (65 kDa)

Caspase 3

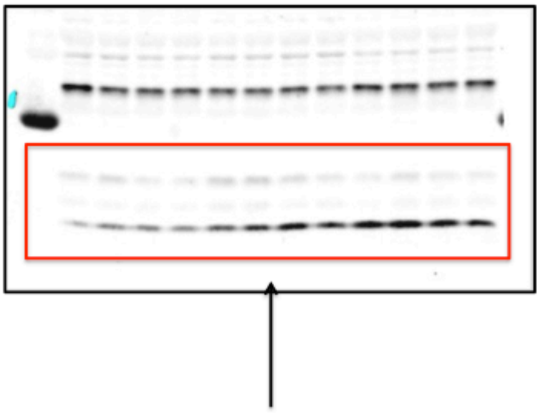

Figure Image (Cropped)
Cleaved Caspase 3 (19, 17 kDa)

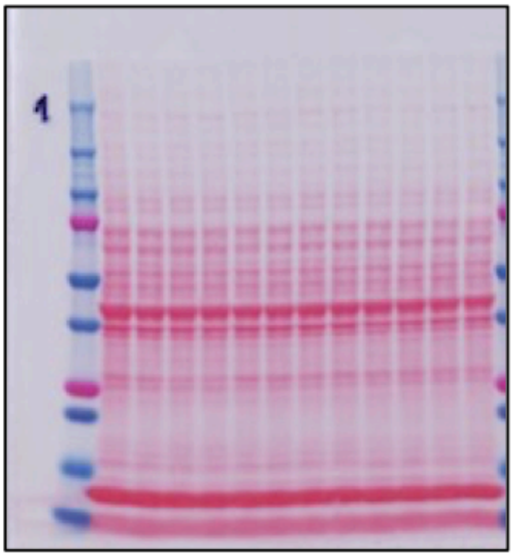

Supplement: Supplementary file 1 — Dataset 1 [file 41598_2018_35809_MOESM1_ESM.pdf]
